# Outcome of severe lactic acidosis associated with metformin accumulation

**DOI:** 10.1186/cc9376

**Published:** 2010-12-20

**Authors:** Sigrun Friesecke, Peter Abel, Markus Roser, Stephan B Felix, Soeren Runge

**Affiliations:** 1 Department of Cardiology, Pneumology and Intensive Care Medicine, Ernst Moritz Arndt University, 17475 Greifswald, Germany; 2 Department of Clinical Chemistry and Laboratory Medicine, Ernst Moritz Arndt University, 17475 Greifswald, Germany; 3 Department of Gastroenterology, Nephrology, Endocrinology, Nutrition, Ernst Moritz Arndt University, 17475 Greifswald, Germany

## Abstract

**Introduction:**

Metformin associated lactic acidosis (MALA) may complicate metformin therapy, particularly if metformin accumulates due to renal dysfunction. Profound lactic acidosis (LA) generally predicts poor outcome. We aimed to determine if MALA differs in outcome from LA of other origin (LAOO).

**Methods:**

We conducted a retrospective analysis of all patients admitted with LA to our medical ICU of a tertiary referral center during a 5-year period. MALA patients and LAOO patients were compared with respect to parameters of acid-base balance, serum creatinine, hospital outcome, Simplified Acute Physiology Score II (SAPS II) and Sequential Organ Failure Assessment (SOFA) score, using Pearson's Chi-square or the Mann-Whitney U-test.

**Results:**

Of 197 patients admitted with LA, 10 had been diagnosed with MALA. With MALA, median arterial blood pH was significantly lower (6.78 [range 6.5 to 6.94]) and serum lactate significantly higher (18.7 ± 5.3 mmol/L) than with LAOO (pH 7.20 [range 6.46 to 7.35], mean serum lactate 11.2 ± 6.1 mmol/L). Overall mortality, however, was comparable (MALA 50%, LAOO 74%). Furthermore, survival of patients with arterial blood pH < 7.00 (*N *= 41) was significantly better (50% vs. 0%) if MALA (*N *= 10) was the underlying condition compared to LAOO (*N *= 31).

**Conclusions:**

Compared to similarly severe lactic acidosis of other origin, the prognosis of MALA is significantly better. MALA should be considered in metformin-treated patients presenting with lactic acidosis.

## Introduction

Metformin is recommended as the treatment of choice in patients with type 2 diabetes mellitus because it decreases cardiovascular morbidity and mortality [1,2]. Nevertheless, metformin-associated lactic acidosis (MALA) is a rare but potentially life-threatening complication with a mortality rate of 30% to 50% [3,4]. The biochemical mechanism of MALA is still not fully understood. Presumably, accumulated metformin suppresses biological oxidation and the enzymes of the citric acid cycle [3,5-7]. Moreover, recent studies have reported that metformin action may be mediated by the regulation of gene expression [8-10]. The pathogenesis of MALA is controversial. MALA is assumed to be triggered by an acute primary tissue hypoxia as in septic shock or cardiovascular failure in most cases [1,3,11,12]. Some authors dispute whether metformin may contribute to lactic acidosis (LA) at all [12,13]. However, cases of MALA solely following drug accumulation have been reported [5,14-16]. Furthermore, data from a US poison center give some evidence that a large single ingestion can lead to LA [17]. Remarkably, severe MALA may have an unexpectedly favorable outcome [4,14,15]. Therefore, the aim of this study was to compare the outcomes of MALA and LA of other origin (LAOO).

## Materials and methods

This retrospective analysis was approved by the institutional ethics committee at Ernst Moritz Arndt University of Greifswald. The necessity of patient consent was waived. All patients admitted to the medical intensive care unit of our tertiary referral center during a 5-year period (2004 to 2008) were screened for LA (that is, lactate of greater than 5 mmol/L and pH of not more than 7.35 [11]) on admission by using the laboratory database.

Clinical characteristics, admission laboratory results, metformin plasma levels, and hospital survival of all LA patients were extracted from patient files. To compare severity of disease, Simplified Acute Physiology Score II (SAPS II) [18,19] and Sequential Organ Failure Assessment (SOFA) score [20,21] were extracted from the ward database. According to discharge diagnoses, patients were categorized under the following entities of LA: MALA, post-cardiopulmonary resuscitation, septic shock, cardiogenic shock, acute mesenteric ischemia, hemorrhagic shock, or other. The diagnosis of MALA was confirmed retrospectively by critical review of patient charts, which included data on medical history, clinical presentation, highly elevated plasma levels of metformin, and absence of other common causes of LA. None of the patients had evidence of hepatic failure. All MALA patients had an electrocardiogram and a bedside echocardiogram to rule out myocardial infarction and relevant cardiac dysfunction. None had evidence of a septic focus in chest x-ray, abdominal or thoracic ultrasound, blood cultures, urinary dipstick (if not anuric), or computed tomography (if indicated). MALA patients were compared with all LAOO patients and with the severely acidotic subgroup (pH < 7.0) of LAOO patients with respect to parameters of acid-base balance, serum creatinine, SAPS II, and hospital outcome by using the Pearson chi-square test or the Mann-Whitney *U *test.

## Results

During the study period, 197 patients were admitted with LA. MALA was diagnosed in 10 patients, none had deliberately overdosed (daily metformin dose of 1,900 ± 356 mg), but all had severe renal failure (median admission serum creatinine of 776 μmol/L, range of 347 to 1,502) that had not been noticed before. In 6 patients, previous diarrhea or vomiting or both were reported; in the other 4, no cause for acute renal failure could be identified, but 3 of them had pre-existing chronic renal insufficiency. Metformin plasma levels were highly toxic in all MALA patients (median of 55 mg/L, range of 31 to 85, therapeutic range of 0.2 to 1.3). All MALA patients were in circulatory shock and were treated with vasopressors. Nevertheless, in 8 of them, circulatory failure deteriorated up to cardiocirculatory arrest, which was reversible with cardiopulmonary resuscitation. All MALA patients were mechanically ventilated and immediately treated with continuous veno-venous hemodialysis or hemofiltration. In MALA, LA and renal dysfunction were significantly more severe, other organ dysfunctions were similar, but mortality was not significantly higher compared with all LAOO patients and with each of the causal entities of LAOO (Table 1).

**Table 1:** Comparison of patients with metformin-associated lactic acidosis with patients with lactic acidosis of other origin

	All patients (*n *= 197)	MALA (*n *= 10)	LAOO (*n *= 187)	Post-cardiac arrest (*n *= 72)	Septic shock (*n *= 42)	Cardiogenic shock (*n *= 39)	Mesenteric ischemia (*n *= 8)	Hemorrhagic shock (*n *= 13)	Other causes (*n *= 13)
Age, years	67 ± 13	66 ± 9	67 ± 13	65 ± 13	68 ± 12	73 ± 11	72 ± 9	64 ± 15	56 ± 14
Male gender	121 (61%)	4 (40%)	117 (63%)	45 (62%)	24 (57%)	26 (67%)	6 (75%)	7 (54%)	9 (69%)
SAPS II	74 ± 20	88 ± 23	73 ± 19^a^	81 ± 16	71 ± 19^a^	75 ± 18	73 ± 25	54 ± 15^b^	54 ± 14^b^
SOFA score	12.5 ± 3.6	14.3 ± 1.4	12.4 ± 3.7	12.9 ± 2.9	12.1 ± 4.5	11.8 ± 2.8^b^	14.4 ± 4.4	13.4 ± 4.6	9.6 ± 4.2^b^
Respiratory	2.5 ± 1.0	2.7 ± 0.9	2.5 ± 1.0	2.7 ± 0.9	2.1 ± 1.3	2.4 ± 0.6	2.6 ± 1.1	2.3 ± 1.5	2.2 ± 1.4
Renal	2.4 ± 1.2	4.0 ± 0.0	2.3 ± 1.2^b^	2.1 ± 1.2^b^	2.5 ± 1.2^b^	2.5 ± 1.1^b^	2.8 ± 0.8^a^	2.1 ± 1.5^b^	1.9 ± 1.3^b^
Hepatic	0.7 ± 1.0	0.3 ± 0.7	0.7 ± 1.0	0.3 ± 0.6	1.3 ± 1.2^a^	0.6 ± 0.8	0.6 ± 0.9	1.8 ± 1.7	0.9 ± 0.9
Cardiovascular	3.3 ± 1.4	4.0 ± 0.0	3.3 ± 1.5	3.5 ± 1.3	3.2 ± 1.5	3.4 ± 1.5	4.0 ± 0.0	2.9 ± 1.8	2.1 ± 2.0^a^
Hematologic	0.9 ± 1.2	0.5 ± 0.8	0.9 ± 1.2	0.6 ± 0.9	1.3 ± 1.4	0.3 ± 0.7	2.2 ± 1.6	2.6 ± 1.1	0.9 ± 1.2
Neurologic	2.7 ± 1.4	2.8 ± 1.5	2.7 ± 1.4	3.7 ± 0.6^a^	1.7 ± 1.3^a^	2.8 ± 1.2	2.2 ± 1.3	1.8 ± 1.4^b^	1.6 ± 1.2
Lactate, mmol/L	11.6 ± 6.2	18.7 ± 5.3	11.2 ± 6.1^b^	12.8 ± 6.1^b^	8.7 ± 4.5^b^	10.9 ± 5.2^b^	8.6 ± 3.3^b^	13.5 ± 9.5^b^	10.8 ± 7.1^b^
pH	7.13 ± 0.19	6.75 ± 0.13	7.15 ± 0.17^b^	7.07 ± 0.19^b^	7.23 ± 0.09^b^	7.18 ± 0.14^b^	7.27 ± 0.04^b^	7.26 ± 0.07^b^	7.14 ± 0.18^b^
Bicarbonate, mmol/L	14.6 ± 5.9	4.4 ± 2.4	15.2 ± 5.6^b^	14.5 ± 5.6^b^	16.6 ± 5.8^b^	14.9 ± 4.0^b^	16.2 ± 1.8^b^	15.8 ± 5.7^b^	14.1 ± 8.8^b^
Creatinine, μmol/L	307 ± 265	796 ± 324	227 ± 141^b^	169 ± 128^b^	318 ± 181^b^	182 ± 48^b^	299 ± 125^a^	303 ± 122^a^	214 ± 57^b^
Survival	52 (26%)	5 (50%)	48 (26%)	19 (26%)	9 (21%)	9 (23%)	0	5 (38%)	6 (46%)

Values are presented as mean ± standard deviation or as number (percentage). Comparison with metformin-associated lactic acidosis (MALA): ^a^*P *< 0.05, ^b^*P *< 0.01. Simplified Acute Physiology Score II (SAPS II) and Sequential Organ Failure Assessment (SOFA) score were measured on admission. Other causes of lactic acidosis were respiratory exhaustion (3), alcoholic lactic acidosis (3), acute liver failure (2), status epilepticus (2), anaphylactic shock, hypovolemic shock, and disseminated intravascular coagulation in acute myeloid leukemia. LAOO, lactic acidosis of other origin.

All MALA patients and 31 of the LAOO patients had very severe acidosis (pH < 7.0). In this subgroup, acid-base imbalance in MALA was still slightly more profound than in LAOO, severity of disease and non-renal organ dysfunction (SAPS II and SOFA score) were comparable, but outcome was significantly better (50% survivors in MALA versus 0% in LAOO) (Table 2).

**Table 2:** Comparison of metformin-associated lactic acidosis and very severe lactic acidosis of other origin (pH < 7.0)

	All patients with pH < 7.0 (*n *= 41)	MALA (*n *= 10)	LAOO (*n *= 31)
Age, years	64 ± 13	66 ± 9	64 ± 14
Male gender	19 (46%)	4 (40%)	15 (48%)
SAPS II	84 ± 18	88 ± 23	83 ± 16
SOFA score	14.0 ± 2.9	14.3 ± 1.4	13.8 ± 3.4
Respiratory	2.7 ± 1.0	2.7 ± 0.9	2.8 ± 1.0
Renal	3.0 ± 1.1	4.0 ± 0.0	2.6 ± 1.1^a^
Hepatic	0.5 ± 0.8	0.3 ± 0.7	0.6 ± 0.9
Cardiovascular	3.6 ± 1.2	4.0 ± 0.0	3.4 ± 1.4
Hematologic	0.8 ± 1.1	0.5 ± 0.8	0.9 ± 1.2
Neurologic	3.3 ± 1.2	2.8 ± 1.5	3.5 ± 1.1
Lactate, mmol/L	18.1 ± 6.5	18.7 ± 5.3	17.9 ± 6.9
pH	6.83 ± 0.12	6.75 ± 0.13	6.86 ± 0.11^a^
Bicarbonate, mmol/L	9.0 ± 4.8	4.4 ± 2.4	10.5 ± 4.5^a^
Creatinine, μmol/L	529 ± 392	796 ± 324	195 ± 91^a^
Survival	4 (10%)	5 (50%)	0 (0%)^a^

Values are presented as mean ± standard deviation or as number (percentage). Comparison with metformin-associated lactic acidosis (MALA): ^a^*P *< 0.01. LAOO, lactic acidosis of other origin; SAPS II, Simplified Acute Physiology Score II; SOFA, Sequential Organ Failure Assessment.

There was no difference within the MALA group between survivors and non-survivors in regard to serum metformin level, the degree of LA, or organ dysfunction (Table 3). MALA patients who did not survive died from refractory circulatory failure after a median of 27 hours (range of 3 to 41 hours) from intensive care unit admission.

**Table 3:** Comparison of survived versus deceased patients with metformin-associated lactic acidosis

	All patients with MALA (*n *= 10)	Survived (*n *= 5)	Deceased (*n *= 5)
Age, years	66 ± 9	64 ± 5	69 ± 11
Male gender	4 (40%)	3 (60%)	1 (20%)
SAPS II	88 ± 23	73 ± 25	103 ± 7
SOFA score	14.3 ± 1.4	13.8 ± 1.5	14.8 ± 1.3
Respiratory	2.7 ± 0.9	2.4 ± 1.1	3.0 ± 0.7
Renal	4.0 ± 0.0	4.0 ± 0.0	4.0 ± 0.0
Hepatic	0.3 ± 0.7	0.4 ± 0.9	0.2 ± 0.4
Cardiovascular	4.0 ± 0.0	4.0 ± 0.0	4.0 ± 0.0
Hematologic	0.5 ± 0.8	0.4 ± 0.9	0.6 ± 0.9
Neurologic	2.8 ± 1.5	2.6 ± 1.3	3.0 ± 1.7
Lactate, mmol/L	18.7 ± 5.3	17.7 ± 5.3	19.8 ± 5.7
pH	6.75 ± 0.13	6.72 ± 0.17	6.77 ± 0.07
Prothrombin activity, %	69.1 ± 27.7^a^	78.0 ± 24.6	58.0 ± 30.7^a^
Serum metformin, mg/L	55 ± 13	52 ± 7	58 ± 19

Values other than 'Male gender', which is presented as number (percentage), are presented as mean ± standard deviation. ^a^One patient was omitted with 11% prothrombin activity due to cumarin therapy. MALA, metformin-associated lactic acidosis; SAPS II, Simplified Acute Physiology Score II; SOFA, Sequential Organ Failure Assessment.

## Discussion

In 10 out of 197 patients in our study, LA on admission was found to be associated with metformin accumulation. MALA patients had the most severe acid-base imbalance but did not have a worse outcome. Mortality rates in our study are in accordance with those published by other authors. For MALA, a mortality of 30% to 50% has been reported [3,4,22]; for LA in general, a mortality of up to 83% has been reported [23,24].

Mortality is generally correlated with lactate levels [24,25]. For MALA, data are less clear. After a retrospective analysis in 1999, Lalau and Race [25,26] reasoned that neither lactate nor metformin levels were of prognostic value in MALA since, in their study, the lactate and metformin levels of survivors were, respectively, similar to and higher than those of patients who died. The authors suppose that the underlying condition, and not metformin accumulation, determined outcome. In contrast, Dell'Aglio and colleagues [27] reported a correlation between pH nadir, metformin level, and outcome.

Recently, observations in two series of MALA were published [4,22]. Peters and colleagues [22] reported 30 patients who were generally less sick than ours (less acidotic, less frequently in shock, lower SAPS II, and less frequently ventilated) and in whom mortality was 30% compared with our 50%. Seidowsky and colleagues [4] reported 42 patients, 29 of whom had incidental metformin accumulation. In these 29 patients, severity of acidosis (mean pH of 6.9) and mortality (48.3%) were comparable to those of our MALA patients [4]. In both series, decreased prothrombin activity on admission was associated with mortality [4,22]. In our small number of cases, we could not reproduce this association.

Compared with our LAOO subgroup with similarly severe acid-base imbalance (pH < 7.0), the outcome of MALA patients was significantly better. Lalau and Race [28] reported decreased mortality for patients who had a pH of less than 7 and who had been treated with metformin (81% versus 99%) and this is consistent with our results.

We suppose that the different outcomes of MALA and LAOO may have been because, unlike most cases of LAOO, LA in MALA is not due primarily to shock or ischemia and because our patients had no underlying condition serious enough to cause severe LA in the absence of metformin accumulation. Furthermore, it has been hypothesized that metformin, possibly through its beneficial effects on vasomotility, might even be protective in shock [26,29].

MALA may develop without an underlying disease or may aggravate LAOO, or metformin may accumulate just coincidentally with LA [3,12]. In any given patient, it will be difficult to establish the causative role of metformin accumulation in the development of LA [4]. Furthermore, the role of metformin in the development of LA is controversial; some authors deny any causal involvement [12,13] altogether, and others believe that metformin actually induces LA in certain cases [30]. Clinical data in general and our retrospective observation in particular are not suitable to decide this issue definitely, but our results and those of other authors point to the latter position [4,5,14-17,22,27]. In our MALA patients, no other cause for LA was identified, and highly elevated metformin plasma levels were measured. In these patients, previously unnoticed deterioration of renal function explained why metformin had accumulated despite having been taken in usual doses. The much greater extent of renal dysfunction in MALA compared with similarly severe LAOO suggests that renal failure not only complicated MALA but also indeed contributed to the pathogenesis. Progressive renal impairment has been recognized as a risk factor for MALA [14,15,30] and cannot be held responsible for LA as a single condition in the absence of metformin [31].

MALA may be more common in severely acidotic patients: 24% of our patients with pH of less than 7 were diagnosed with MALA. Therefore, given the unexpectedly favorable prognosis, it appears important to consider MALA and look into the possibility of metformin therapy when a patient is admitted with very severe LA. It may be safely assumed that, in such severely ill patients, only prompt and rigorous therapy allows this favorable prognosis [14]. Anecdotal evidence suggests that high-volume renal replacement therapy may be beneficial in severe MALA [5,32,33].

### Limitations

Our study included a limited number of patients. The incidence of MALA is low, and therefore recruitment of patients is demanding. In this study, we included 10 patients seen in one institution during a 5-year period. A second limitation is the retrospective design. A bias in patient selection cannot be excluded. As metformin levels were usually not determined in cases in which alternative causes of LA had been identified, coincident metformin accumulation may have been missed. To our knowledge, though, this is the first direct comparison between MALA and LAOO.

## Conclusions

The outcome of very severe MALA (pH < 7) is much better than might be expected from the comparison with similarly severe LAOO. To ensure adequate therapy, it appears important to consider an association with metformin and look into the possibility of metformin medication in all cases of very severe LA.

## Key messages

• Given the same profound level of acidosis, prognosis of metformin-associated lactic acidosis (MALA) is significantly better than that of lactic acidosis of other origin.

• It is important to consider MALA in any metformin-treated patient and to start therapy promptly.

## Abbreviations

LA: lactic acidosis; LAOO: lactic acidosis of other origin; MALA: metformin-associated lactic acidosis; SAPS II: Simplified Acute Physiology Score II; SOFA: Sequential Organ Failure Assessment.

## Competing interests

The authors declare that they have no competing interests.

## Authors' contributions

S Friesecke conceived of the study and helped to draft the manuscript. PA participated in the design of the study and performed the statistical analysis. MR carried out data collection and data analysis. S Felix participated in the design and coordination of the study. SR participated in the design of the study and drafted the manuscript. All authors read and approved the final manuscript.
